# Postural balance and functional independence of elderly people according to gender and age: cross-sectional study: Equilíbrio postural e independência funcional de idosos de acordo com o sexo e a idade: estudo transversal

**DOI:** 10.1590/1516-3180.2016.0325280217

**Published:** 2017-04-03

**Authors:** Helen Benincasa Nakagawa, Juliana Rizzatto Ferraresi, Melina Galetti Prata, Marcos Eduardo Scheicher

**Affiliations:** I PT. Physiotherapist, Department of Physiotherapy and Occupational Therapy, Universidade Estadual Paulista “Júlio de Mesquita Filho” (Unesp), Campus de Marília, Marília (SP), Brazil.; II PT. Professor, Department of Physiotherapy, Faculdade Marechal Rondon, São Manuel (SP), Brazil.; III PT, Msc. Physiotherapist, Hospital das Clínicas, Faculdade de Medicina de Marília, Marília (SP), Brazil.; IV PT, PhD. Adjunct Professor, Department of Physiotherapy and Occupational Therapy, Universidade Estadual Paulista “Júlio de Mesquita Filho” (Unesp), Campus de Marília, Marília (SP), Brazil.

**Keywords:** Aging, Postural balance, Activities of daily living, Accidental falls, Drug therapy

## Abstract

**CONTEXT AND OBJECTIVE:**

Aging causes changes in men and women. Studies have shown that women have worse postural balance and greater functional dependence than men, but there is no consensus regarding this. The aim of this study was to compare the balance and functional independence of elderly people according to sex and age, and to evaluate the association between postural balance and the number of drugs taken.

**DESIGN AND SETTING:**

Cross-sectional at a state university.

**METHODS:**

202 elderly people were evaluated regarding balance (Berg Scale), independence (Barthel Index), age, sex, number of medications and physical activity.

**RESULTS:**

The subjects comprised 117 women (70.2 ± 5.6 years old) and 85 men (71.1 ± 6.9 years old). For balance, there was no significant difference regarding sex, but there was a difference regarding age (P < 0.0001). For functional independence, there was a difference regarding sex (P = 0.003), but not regarding age. The variables of age, medications and physical activity were significant for predicting the Berg score. For the Barthel index, only age and sex were significant. Elderly people who took three or more medications/day showed higher risk of falling than those who took up two drugs/day (odds ratio = 5.53, P < 0.0001, 95% confidence interval, 2.3-13.0).

**CONCLUSIONS:**

There was no sexual difference in relation to postural balance. However, people who were more elderly presented a high risk of falling. Functional dependence was worse among females. There was an association between the number of medication drugs and risk of falling.

## RESUMO

**CONTEXTO E OBJETIVO:**

O envelhecimento provoca alterações em homens e mulheres. Estudos mostraram que mulheres têm pior equilíbrio postural e maior dependência funcional do que homens, mas isso não é consenso. O objetivo deste estudo foi comparar o equilíbrio e a independência funcional de idosos por sexo e idade, e avaliar a associação de equilíbrio postural com o número de medicamentos ingeridos.

**TIPO DE ESTUDO E LOCAL:**

Estudo transversal em uma universidade estadual.

**MÉTODOS:**

202 idosos foram avaliados quanto ao equilíbrio (Escala de Berg), independência (Índice de Barthel), além da idade, sexo, número de medicamentos e atividade física.

**RESULTADOS:**

Os sujeitos foram 117 mulheres (70,2 ± 5,6 anos) e 85 homens (71,1 ± 6,9 anos). No equilíbrio, não houve diferença significativa por sexo, mas houve por idade (P < 0.0001). Para a independência funcional, houve diferença por sexo (P = 0,003), mas não por idade. As variáveis idade, medicação e atividade física foram significativas para predizer o escore de Berg. Para o índice de Barthel, apenas a idade e o sexo foram significativos. Idosos que ingeriram 3 ou mais medicamentos/dia mostraram maior risco de cair do que os que ingeriram até 2 drogas/dia (*odds ratio* = 5,53, P < 0.0001, intervalo de confiança de 95%, 2,3-13,0).

**CONCLUSÕES:**

Não houve diferença entre sexos em relação ao equilíbrio postural; idosos mais velhos, contudo, apresentaram grande risco de cair; a dependência funcional foi pior no sexo feminino. Foi encontrada associação entre número de medicamentos utilizados e risco de cair.

## INTRODUCTION

The global population is aging at an unprecedented rate. In 2012, 23% of the population in the more developed regions and 9% in the less developed regions were aged 60 years or over.^1^ It has been estimated that by 2050, the proportion of older citizens will increase to 32% in developed countries and 19% in developing countries.^1^

Human aging causes physiological changes such as decreased postural balance, thus increasing the risk of falls. Postural control is considered to be a complex motor skill derived from interaction of multiple sensorimotor processes.^2^ Age-related changes in the peripheral and central components of the visual, somatosensory and vestibular systems can be expected to affect balance and mobility.

One-third of people aged 65 years and over fall one or more times a year. Among community-dwelling older people, the cumulative incidence of falls ranges from 25 to 40%.^3^ Falls have been correlated with a number of different risk factors. Some of these, like age or sex, cannot be altered. In a review, Meschial et al.^4^ found contradictory results in several databases concerning the proportion of falls with regard to sex. Four studies reporting that women were mostly affected were identified, while one study indicated that men were more prone to falling.

Prospective cohort studies have indicated that falls seem to be an independent determinant of functional decline and dependency in activities of daily living (ADLs) in a general elderly population.^5^^,^^6^ Sposito et al. showed that women have higher dependence than men in carrying out activities of daily living.^7^

Aging causes the appearance of chronic diseases, and consequently there is an increase in the quantity of medication drugs ingested.^8^ Both specific classes of drugs and the total number of drugs may be associated with imbalance^9^ and dependency in activities of daily living.

## OBJECTIVE

The objectives of this study were to compare the balance and level of functional independence of older adults according to sex and age, and to evaluate the association between postural balance and number of drugs taken.

## METHODS

### Design, participants and ethics

**Figure 1:**
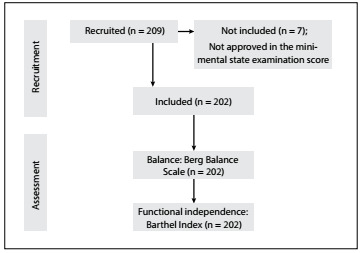
Procedures for data collection.

A cross-sectional study was conducted between 2009 and 2013 in the city of Marília, São Paulo, Brazil. A convenience sample of 209 community-dwelling elderly individuals was recruited at two basic healthcare public units, four healthcare public centers, five community centers and two geriatric clinics. Of the 209 participants initially recruited, 7 were withdrawn because they did not meet the inclusion criteria. Thus, a total of 202 elderly people were enrolled in the study. The study design can be seen in **Figure 1**. The following inclusion criteria were used: age 60 years or older; living in the community; and independent gait (without gait assistance device). The following exclusion criteria were used: cognitive impairment detectable by means of the mini-mental state examination (MMSE), with the following cutoffs: 20 for illiterates; 25 for schooling level of 1 to 4 years; 26.5 for 5 to 8 years; 28 for 9 to 11 years; and 29 for higher levels of education;^10^ and factors that interfere with corporal balance, such as: sequelae of neuromusculoskeletal diseases (stroke or Parkinson’s disease), uncorrected visual problems, orthostatic hypotension and continuous use of sedatives, antidepressants and hypnotics. The elderly subjects were classified as sedentary or active according to the criteria of the Brazilian Society of Sports Medicine and the Brazilian Society of Geriatrics and Gerontology.^11^

Written informed consent was obtained from all patients before enrollment. The study was submitted and approved by the Research Ethics Committee of the School of Philosophy and Sciences, Universidade Estadual Paulista (UNESP), Marília, São Paulo, Brazil. It was carried out in accordance with Resolution no. 196/96 of the National Health Council.

### Outcome measurements

Data were collected via face-to-face interviews with researchers. Within the scope of the present study, the subjects were asked for demographic information such as age, diseases presented and medications used.

The participants were evaluated regarding their balance using the Berg Balance Scale (BBS) and their functional independence in daily activities was evaluated using the Barthel Index (BI).

#### Balance

The BBS, which measures “functional balance,” has three dimensions: maintenance of a position, postural adjustment to voluntary movements and reaction to external disturbances. The subjects’ performance in each of 14 activities is measured on a five-point ordinal scale ranging from 0 to 4 (0 = unable to perform; 4 = independent), such that the aggregate score ranges from 0 to 56. Scores of 48 and less indicate inability to walk independently and safely in daily life and a greater risk of falls.^12^

#### Functional independence in daily life

The BI is a reliable and valid tool measuring overall disability that represents a subject’s ability to perform tasks within activities of daily living. It has been recommended for functional assessments on older people. The version used here evaluated functional independence in relation to 10 activities: feeding, bathing, grooming, dressing, bowel care, bladder care, toilet use, transfers, walking and stair climbing. The overall score is obtained by attributing points to each category, depending on the time taken and assistance needed by each patient. The score ranges from 0 to 100, in 5-point intervals, and the higher the score is, the more independent the patient is.^13^

### Data analysis

The Kolmogorov-Smirnov test was used to determine the data distribution. The Mann-Whitney test was used for comparisons between the genders. Correlations between drugs used and the BBS were made using the chi-square test (with Yates correction), with the cutoff point ≤ 48 in BBS for greater risk of falls.^12^ Comparisons between the subjects’ ages were made using one-way ANOVA with Dunn’s post-test. To analyze the effect of independent variables on the dependent variable (Berg or Barthel), a multiple linear regression model was constructed by means of the Enter method (forced input). R2 was analyzed to ascertain the coefficient of determination of the percentage variation explained by the model. ANOVA for repeated measurements was used to compare the Berg and Barthel scales; however, in order to analyze the influence of factors such as age, sex, medication and physical activity, these were included as covariables (ANCOVA). Furthermore, in order to control for the effect of covariables regarding the correlation analysis between Berg and Barthel, a partial correlation analysis was performed. Pearson’s correlation test was performed to analyze the correlation without controlling for covariables. The data were analyzed using the SPSS software, version 19.0 for Windows, and P ≤ 0.05 was accepted as significant.

## RESULTS

Among the 202 elderly people studied, 117 were women (57.9%) and 85 were men (42.1%). With regard to schooling level, 12 participants (5.94%) were illiterate, 71 (35.14%) had attended school for 1-4 years, 43 (21.32%) for 5-8 years and 76 (37.62%) for more than 8 years. **Table 1** shows the characteristics of the subjects of this study.

**Table 1:**
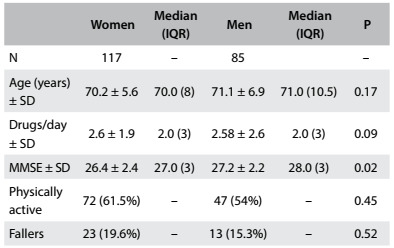
Subjects’ characteristics

SD = standard deviation, MMSE = mini-mental state examination, IQR = interquartile range.

The BBS scores showed significant differences between the age groups (60-69 years, 70-79 years and ≥ 80 years), with lower scores in older age groups. For the Barthel index, there was no significant difference (**Table 2**), although in older age groups, the scores were lower, thus indicating greater reliance in the subjects’ activities.

**Table 2:**
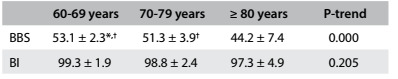
Comparison of scores on Berg Balance Scale (BBS) and Barthel Index (BI), according to age groups

Values are the mean ± standard deviation; *P < 0.01 in relation to age 70-79 years, †P < 0.001 in relation to age ≥ 80 years.

The Berg and Barthel scales showed a significant positive correlation. When the variables (age, sex, medication and physical activity) were controlled for, the correlation strength was lower, thus indicating that these variables had an important effect (**Table 3**).

**Table 3:**
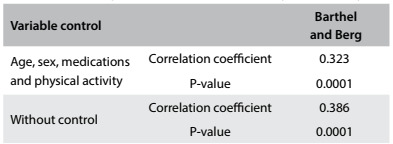
Correlation analysis with and without controlling for the variables of age, sex, medications and physical activity

**Figure 2:**
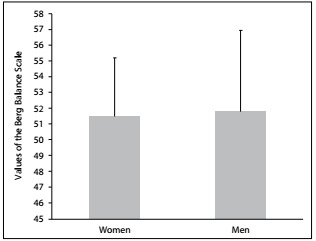
Comparison of scores on Berg Balance Scale (BBS) between women (n = 117; BBS = 51.5 ± 4.3) and men (n = 85; BBS = 51.8 ± 3.3); P = 0.08.

The degree of balance was found to be lower among the women than among the men (51.5 ± 4.3 and 51.8 ± 3.3, respectively; P = 0.08). There was no significant difference in postural balance between the sexes (**Figure 2**).

**Figure 3:**
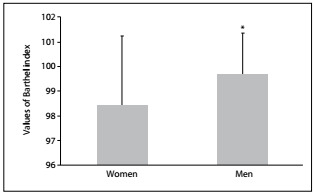
Comparison of scores in Barthel index (BI) between women (n = 117; BI = 98.4 ± 2.9) and men (n = 85; BI = 99.7 ± 1.7); *P = 0.003.

**Figure 3** presents the Barthel scale and shows that the men had a higher average score (99.7 ± 1.7) than the women (98.4 ± 2.9), thus indicating greater dependence among the women than among the men in the tasks evaluated by the scale (P = 0.003). The regression analysis confirmed these data and showed that being a woman contributed towards having a worse Barthel index score (**Table 4**).

**Table 4:**
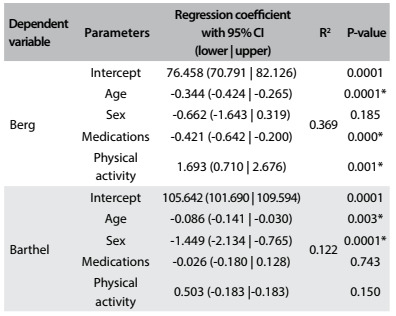
Multiple linear regression to analyze the effect of the independent variables on the Berg and Barthel scales

*Significant effect of the variable in the regression model for predicting the dependent variable. CI = confidence interval.

There was an association between the number of drugs taken and the risk of falling. Elderly people who reported ingesting three or more medications/day presented higher risk of falling than those who reported taking up to two drugs/day (odds ratio = 5.53; P < 0.0001; 95% confidence interval, CI: 2.3-13.0), considering a cutoff ≤ 48 points for higher risk of falls on the Berg scale.^12^

Through regression analysis, it could be seen that the variables of age, medications and physical activity significantly predicted the Berg score. In addition, the set of variables inserted in the model explained 36.9% (R^2^) of the variation in the Berg scores. For the Barthel index, only age and sex were significant, but the regression model indicated that the variables together account for only 12.2% (R^2^) of the variation of the scores. This indicates that the Barthel score appears to be less influenced by the covariates of age, sex, medications and physical activity than the Berg scale (**Table 4**).

## DISCUSSION

This study examined some important issues regarding elderly people, including the difference between sex and age groups regarding balance and independence, and the relationship between the risk of falling and the number of drugs ingested. There were no differences in postural balance in relation to sex (**Figure 2** and **Table 4**). These results are contrary to data in the literature. Perracini and Ramos^14^ and Moreira et al.^15^ indicated that women had worse balance than men. The probable reason for our result is that more than 50% of the elderly people in the sample evaluated here practiced regular physical activity (61.5% for women and 54% for men). The literature provides evidence that older adults who maintain regular physical activity have better postural balance and mobility.^16^^,^^17^^,^^18^ Comparison among age groups, regardless of sex, showed significant differences in BBS scores, thus indicating that deterioration of balance is associated with advancing age and that balance among people aged 80 and over is impaired (mean score 44.2), with high risk of falling (**Table 2** and **Table 4**). The deterioration of postural control with age can be explained by decreased cognitive function, declining sensory inputs and motor responses and deterioration of the integration of systems responsible for postural balance.

The Berg and Barthel scales showed a significant positive correlation, but when the variables of age, sex, medication and physical activity were controlled for, the strength of the correlation became lower, thus indicating that these variables had an important effect (**Table 3**). The variables of age, medications and physical activity significantly predicted the Berg score, thus explaining 36.9% (R^2^) of the variation in the Berg scores. For the Barthel index, only age and sex were significant, but the regression model indicated that the variables together were responsible for only 12.2% (R^2^) of the variation of the scores. This indicated that the Barthel score appeared to be less influenced by the covariates of age, sex, medications and physical activity than the Berg scale. This data are in contrast with the study of Dunlop al.,^19^ which found a strong relationship between activities of daily living (ADL) and level of physical activities, age and female gender in older adults.

The present study also examined the relationship between the number of drugs taken and balance, and found that older adults who take three or more drugs/day are at higher risk of falls, according to the Berg scale. The number of drugs taken daily has been identified as an independent risk factor for falls: Campbell et al. found that the total number of drugs was an important predictor of falls among women.^20^

The literature indicates that sex is a factor strongly related to occurrences of functional dependency, and that the chance that women will be dependent is twice as high as for men.^21^^,^^22^^,^^23^^,^^24^ Our results showed that men achieved scores that were higher than those of women on the Barthel Index, thus indicating a greater likelihood that women are more dependent than men in relation to activities of daily living (**Figure 3** and **Table 4**). This difference can be explained through two issues: the predominance of non-lethal incapacitating conditions among women (osteoarthritis, osteoporosis and depression) and the greater capacity of women to report their health status than that of men of the same age.^21^ Women have longer life expectancy than men,^1^ thus increasing their risk of chronic diseases, which can lead to disability. Furthermore, women tend to report greater functional difficulties than men.^25^

Analysis on the functional capacity of elderly people can be considered an essential mechanism for more detailed clinical evaluation in the field of gerontology and rehabilitation^22^ as well as for research relating to postural balance.

One of the limitations found in the present study was the difficulty in finding male elderly individuals who were willing to participate and thus being able to make comparisons with equal numbers of men and women. The strengths of this study were the number of participants and the tests used in evaluations, which are easy to apply in clinical practice.

## CONCLUSIONS

The results showed that there was no difference between the two sexes in relation to postural balance in the population studied. However, the older age group presented a great risk of falling. Functional dependence was correlated to sex, such that it was worse among females. Furthermore, there was an association between the number of drugs taken and the risk of falling.
